# Hypoperfusion in Supramarginal and Orbital Gyrus, Position Discrimination Test, and Microsaccades as a Predictor of Pisa Syndrome in Parkinson's Disease

**DOI:** 10.1155/2024/5550362

**Published:** 2024-05-30

**Authors:** Asako Yoritaka, Tetsuo Hayashi, Keiko Fusegi, Sachiko Nakayama, Jun Haneda, Nobutaka Hattori

**Affiliations:** ^1^Department of Neurology, Juntendo University Koshigaya Hospital, Saitama 343-0032, Japan; ^2^Department of Radiology, Koshigaya Municipal Hospital, Saitama 343-8577, Japan; ^3^Department of Neurology, Juntendo University School of Medicine, Tokyo 113-8421, Japan

## Abstract

Patients with Parkinson's disease (PD) experience significantly reduced quality of life when PD is complicated with Pisa syndrome (PS). PS is a postural abnormality associated with a lateral bending of the trunk, causing the patient to lean to one side. Microsaccades during fixation are transmitted to the visual cortex, and this gaze movement may be impaired in PD. We aimed to detect presymptomatic signs of PS. We enrolled 50 patients with PD without dementia and investigated the visual systems in patients with concurrent PD and PS based on a Romberg ratio of<1.0. Gaze analysis, pupil diameter, stabilization tests, neuropsychological tests, and cerebral perfusion scintigraphy were reviewed and statistically analyzed. Two years later, we divided the patients into three groups as follows: PISA++ (patients who had PS at enrollment), PISA-+ (patients without PS that developed PS during the 2-year period), and PISA-- (patients without PS that did not develop PS during the 2-year period). The PISA-+ group exhibited a significantly higher daily levodopa dose and longer fixations, as well as lower position discrimination, Wechsler Adult Intelligence Scale-Third Edition blocking, and blood flow in the left supramarginal and orbital gyri than that in the PISA-- group. The PISA++ group showed a significantly longer fixation time and lower Mini-Mental State Examination score, Romberg ratio of area, amplitude, velocity of microsaccades, and blood flow in the left precuneus and cuneus than that in the PISA-+ group. Before the onset of PS, hypoperfusion occurred in the correlative visual cortex and the position discrimination test. Patients with PS have reduced saccades and slow microsaccades.

## 1. Introduction

Dopamine replacement with levodopa or dopamine agonists markedly improves motor symptoms, disability, and prognosis in patients with Parkinson's disease (PD) [1, 2]. However, levodopa use is associated with the development of motor complications, such as dyskinesia and wearing-off in patients with advanced PD, substantially contributing to overall disability and reduced quality of life [3]. Patients with PD experience critical inconvenience when PD is accompanied by Pisa syndrome (PS), characterized by distinct lateral bending [4] of the trunk, causing the patient to lean to one side.

In previous studies, patients with concurrent PD and PS were associated with altered attention and visuo-perceptual functions [4, 5], suggesting alterations in the frontal-striatal systems and posterior cortical areas [6]. Posture is controlled by the vestibular, visual, and proprioceptive systems. Patients with PD show signs and symptoms of altered orientation on the vertical axis related to abnormalities in posture, postural instability, and visuospatial vertical perception, vertically and horizontally [7].

Using stabilometry, we found that patients with concurrent PD and PS exhibited the paradox phenomenon (<1.0) of the Romberg rate indicating that postural sway was improved by removing visual perception. The Romberg rate is the ratio of the area or length of the locus of postural sway on the closing eye/opening eye. Disturbance of the visual system is considered crucial in PS. During visual fixation, the eyes are never completely still and produce small involuntary movements called “fixational eye movements,” including microsaccades, drift, and tremors. Microsaccades are the fastest eye movement during fixation. They contribute to maintaining vision during fixation by shifting the retinal image in a fashion that overcomes adaptation, generating a neural response to stationary stimuli in the visual neurons [8–10]. Deviation from the typical horizontal microsaccade direction is related to cognitive impairment in patients with mild cognitive impairment and Alzheimer's disease, and changes in microsaccade direction in these patients are related to specific attentional deficiencies [11, 12].

We investigated the crucial role of the visual system through stability tests, eye-tracking, cognitive function tests, and cerebral blood flow using single-photon emission computed tomography (SPECT). We predicted the subsequent lateral bending of the body.

## 2. Materials and Methods

This prospective study was conducted at the Department of Neurology, Juntendo University, Koshigaya Hospital. Participants were recruited from the outpatient clinic. The study adhered to the Declaration of Helsinki and was approved by the Juntendo University Koshigaya Hospital Institutional Ethics Committee (Juntendo Koshigaya 2019-10). All patients provided written informed consent.

### 2.1. Patients with PD

The inclusion criteria were diagnosis of PD conforming to the Movement Disorder Society (MDS) criteria [13], modified Hoehn and Yahr stage <2.5, and absence of dementia (Mini-Mental State Examination (MMSE) score ≥25).

The exclusion criteria were as follows: disturbance of visual acuity and field; stroke episode; lumbar or hip joint disorder; treatment with deep brain stimulation or levodopa carbidopa intestinal gel; neck tremor or dyskinesia score >2 points on the MDS-Unified Parkinson's Disease Rating Scale (UPDRS); and presence of gait freezing (owing to the potential association with a vestibular perceptive deficit) [14].

### 2.2. Evaluation

The age of onset, sex, disease duration, onset side, anti-Parkinsonian drug, levodopa equivalent dopamine daily dose [15], and MDS-UPDRS Part III were evaluated. Side and back images of the participants were captured using a digital camera. Similarly, the perpendicular line used as a reference was photographed. The patients stood adjacent to the perpendicular line, and the image was taken from a distance of 3 m at half the patient's height. Using the captured images, the angle formed by the vertical line and the line that intersects at 90° with the line connecting the left and right acromions was defined as the lateral bending angle. In our study, we defined “PISA+” as when the angle of lateral bending was >3° because we aimed to focus on the factors that characterize early lateral bending. As this study targeted early lateral bending, we set the cutoff value of the perceived bending at a smaller angle than the specialist consensus of 5° of lateral trunk flexion [16] because truncal bending can sometimes occur rapidly [17]. In our study, participants with lateral bending >3° did not improve their angle.

The assessments of cognitive function were as follows: MMSE; Montreal Cognitive Assessment Japanese version; Dot Counting, Position Discrimination, Number Location, and Cube Analysis tests of the Visual Object and Space Perception Battery (Thames Valley Test Company); clock drawing test (Freedman method); line orientation of Repeatable Battery for the Assessment of Neuropsychological Status; and block design of the Wechsler Adult Intelligence Scale-Third Edition.

To assess postural sway using a gravicorder (GW-31, ANIMA, Inc., Tokyo, Japan), patients were asked to maintain an unperturbed and upright stance of 5 cm (heel-to-heel) with their eyes open and closed (1 min for each action).

Gaze movements and pupil diameters were recorded using a video-based eye-tracking system (Tobii Pro Spectrum 1200, Tobii, Stockholm, Sweden) with a sampling rate of 1200 Hz. The participants sat at a distance of 65 cm from the monitor (23.8 inches, 1080 × 1920 pixels) and observed six scenes for 3s each after six points of calibration. The luminance in the sitting position was 1400 lx. We used the algorithm proposed by Eagel and Kliegl to detect microsaccades [11]. Microsaccades were defined as eye movements <1° in amplitude. The data were recorded binocularly.

SPECT was used to measure the regional cerebral blood flow (rCBF) using *N*-Isopropyl-4-Iodoamphetamine (^123^I) Hydrochloride Injection (^123^I-IMP; Nihon Medi-Physics Co., Tokyo, Japan) at enrollment. Subsequently, 25 min after the 111 MBq intravenous injection, SPECT was performed using a GE Infinite VC detector system (GE Healthcare, Chicago, IL, USA). The Statistical Parametric Mapping-2 (SPM2) (UCL, London, UK) was used for image manipulation. The Digital Imaging and Communications in Medicine files were converted to an analyzable format using freeware software to make them SPM compatible. rCBF differences between the groups were assessed using SPM analysis. A significantly decreased blood flow of IMP was defined by a *Z* score ≥2.5. The SPECT results of the participants were spatially transformed into a Talairach Atlas [18].

Statistical analyses were two-sided, and the level of significance was set at *p* < 0.05 using SPSS version 29 (IBM Corp., Armonk, NY, USA).

## 3. Results

We enrolled 50 patients with PD at baseline. After 2 years of observation, we divided the patients into three groups: PISA++ (*n* = 20), comprising patients with signs of PISA+ at enrollment who continued to experience symptoms throughout the study; PISA-+ (*n* = 9), which included patients without PISA + at enrollment who developed signs of PISA+ during the 2-year study period; and PISA-- (*n* = 21), comprising participants without PISA+ signs who did not develop PISA+ throughout the study. Regarding the side of bending, among the 20 patients with right-sided dominance onset, 5 bent to the right, 2 bent to the left, and 13 did not bend. Among the 17 patients with left-sided dominance, 11 bent to the right side, 3 to the left side, and 3 exhibited no bending. Of the 13 patients with symmetrical onset, 5 bent to the right side, 2 to the left, and 6 showed no bending. The left-sided dominant onset was associated with a higher incidence of PISA+ than the right-sided dominant onset (*p* = 0.0089). The clinical findings of the enrolled patients and the three groups after the follow-up period are shown in Tables 1 and 2, respectively. The number of saccades in the area of interest was 0.35 ± 1.45 and 0.22 ± 1.02 in patients treated with and without levodopa, respectively (*p* < 0.01).

**Figure 1:**
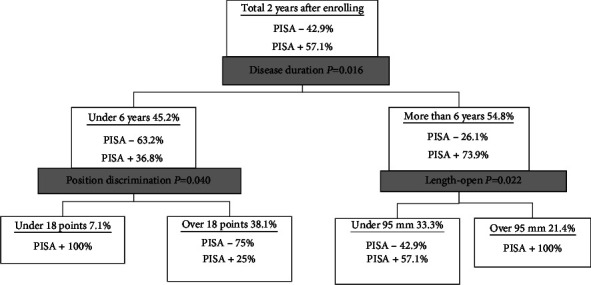
Decision tree model to optimize the PISA+ using the clinical findings at enrollment. The combination of disease duration >6 years and length during eye opening (gravicorder) >95 cm resulted in PISA+. A combination of disease duration <6 years and position discrimination test score <18 points resulted in PISA+.

The decision tree revealed that disease duration was the first vital factor. Patients with a duration of under 6 years and those with a score of <18 points in the position discrimination test had PISA+. Patients with a duration of more than 6 years and those with a longer total trajectory length had PISA+ (Figure 1).

**Figure 2:**
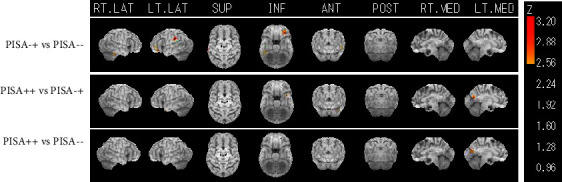
^123^I-IMP single-photon emission computed tomography (SPECT) findings of the three groups. 3DSSP was used to compare hypoperfusion among the three groups. The SPECT images from the participants were spatially transformed into a Talairach Atlas. Significant differences were defined by *Z* scores ≥2.5. PISA-+ vs. PISA-- shows hypoperfusion in the left supramarginal and orbital gyri. PISA++ vs. PISA-+ shows hypoperfusion in the left precuneus, cuneus, and superior temporal gyrus. PISA++ vs. PISA-- shows hypoperfusion in the left precuneus and cuneus.

^123^I-IMP SPECT findings of the three groups revealed that patients with PD exhibited some hypoperfusion in the supramarginal (Brodmann 40) and frontal orbital cortices (Brodmann 11) before lateral bending (Figure 2). Patients with PISA+ exhibited lower perfusion in the precuneus and cuneus.

## 4. Discussion

**Figure 3:**
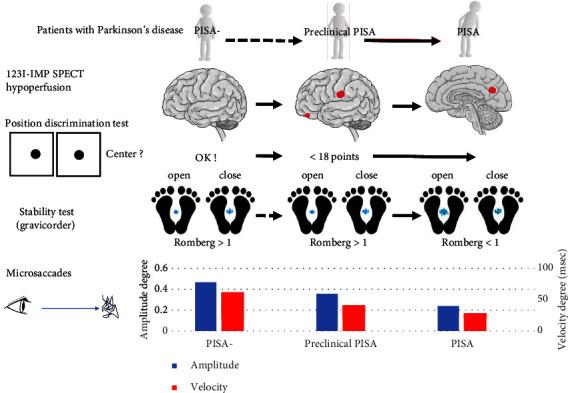
Clinical model of Pisa syndrome in patients with Parkinson's disease. In the stage of preclinical Pisa syndrome, perfusion of the frontal orbital gyrus and supramarginal area and position discrimination score were decreased. In Pisa syndrome, perfusion of the precuneus and cuneus was decreased, and the Romberg rate of the area was <1.0. The amplitude and velocity of microsaccades were decreased in Pisa syndrome. SPECT, single-photon emission computed tomography.

We discovered for the first time that eye-tracking measurements, stability tests, and position discrimination tests could be used as presymptomatic screening tools for lateral bending outcomes (Figure 3). These tests are easily administered and cost-effective. Hypoperfusion in the orbitofrontal and supramarginal cortices, disturbance of visuospatial cognition, and longer fixation preceded the lateral bending in PD. In the presymptomatic period of lateral bending, visuospatial processing and addressing challenges may be affected. When lateral bending occurred, the patients exhibited slow and small microsaccades, decreased saccades, and hypoperfusion of the cuneus and precuneus. The change in visuospatial ability was specific to lateral bending. In contrast, camptocormia was not accompanied by visuospatial deficits [5]. In our previous study on camptocormia, the mean Romberg rate in the affected cases was 1.157 [19], which differed insignificantly from that in this study.

The orbitofrontal gyrus, a presymptomatic lesion observed in this study, is an essential center for processing visual, spatial, and emotional information in surgery for frontal lobe tumors [20]. A bundle to the brainstem connections of the orbitofrontal cortex travels to the caudate, medial to the internal capsule, with radiations to the parietal and occipital lobes traveling with the inferior front-occipital fasciculus [20]. In 50 poststroke lateropulsions, the inferior parietal lobe at the junction of the postcentral gyrus (Brodmann area 2) and supmarginal gyrus Brodmann area 40 was mapped as the most relevant lesion location for lateropulsion [21]. The postcentral gyrus, surrounding white matter, and the postcentral gyrus and Brodmann area 40 in the inferior parietal lobe are reportedly associated with lateropulsion [21–23]. In addition, damage to the inferior frontal and precentral gyri is reportedly associated with lateropulsion [21, 23, 24]. The supramarginal gyrus was believed to be essential in integrating perception with the orbitofrontal cortex, precuneus, and cuneus to maintain the upright position in this study.

The anterior portion of the brain is prone to the posterior portion in free water [25] and iron accumulation imaging [26], which are sensitive to the early stage preceding neuronal degeneration in PD. Hypoperfusion may be observed in the frontal and parietal cortices, followed by the visual cortex. The precuneus is associated with higher-order cognitive function, particularly complex visuospatial processing [27]. The major subcortical connections of the precuneus are the superior colliculus (SC) and nucleus reticularis pontine tegmentum [27]. No differences were observed in the line orientation among the three groups in our study. The judgment of line orientation revealed that the disturbance indicated decreased cerebral blood flow in the bilateral parietal lobe in patients with PD [28] and is a biomarker of PD progression [29].

Conversely, in the dual task performed on one standing leg, participants who achieved a high score on the cognitive tasks showed increased oxygenated hemoglobin levels in the dorsolateral prefrontal cortex and less postural sway than those who scored low [30]. According to the Braak model, neurodegeneration of the pedunculopontine nucleus occurs early in stage 3, whereas Lewy bodies occur later in the neocortex [31]. Disorganization of the frontal eye field pedunculopontine nucleus in patients with PD may be related to a dysfunction of the pedunculopontine nucleus area and not to frontal eye field neurodegeneration because this cortical structure may be spared [31].

Based on gaze analysis, the relationship between microsaccades and the firing of cells in the primary visual cortex (V1) is considered essential [8]. Martinez-Conde et al. tracked eye movements and recorded them from V1 cells as macaque monkeys fixated [8]. When an optimally oriented line is centered over a cell's receptive field, activity increases after microsaccades [8]. Microsaccades and neural markers of covert spatial attention are functionally correlated, and directional biases in microsaccades are correlated with neural signatures of spatial attention [32]. Premotor neurons in the brain stem reticular formation are active during microsaccades [33], demonstrating that voluntary saccades and fixational microsaccades share the same neural mechanism [34]. A recent study found that microsaccades modulate neuronal activity and visually induce gamma-band (30–100 Hz) synchronization in the primate areas of the visual cortex: V1 and V4 [35]. Microsaccades depend on the variability of rostral SC activity [36], which might explain the relationship between changes in microsaccade rates and visibility [10]. Microsaccades, reaction time, and saccade velocity are associated with neural activity in the SC [37]. Saccade, pursuit metrics, blinks, and pupil dilatation were intimately associated with dopamine activity, and the oculomotor function of the basal ganglia is primarily performed by the particular substantia nigra reticularis (SNr) neurons projecting to the SC [38]. When a stable high-valued object appears, SNr neurons are largely inhibited; therefore, SC saccadic neurons are disinhibited, facilitating saccades to the object [39]. Increased basal ganglia inhibition of the SC in PD might cause the decoupling of action and perception [39].

Raw and task-evoked pupil sizes were associated with activity in the locus coeruleus (LC) [40, 41]; therefore, the participant's pupil size was demonstrated to be correlated with the LC-norepinephrine system and was considered a parameter of visual attention. However, in our study, pupil size and change in diameter differed insignificantly between the groups.

Davidsdottir et al. reported that patients with dominant symptoms on the left side were more visually dependent than those with dominant symptoms on the right side, and the parietal-mediated perception of visual space was affected in PD [7]. Lateral bending exhibited a greater left-sided dominant onset than right-sided onset in our study.

This study had some limitations. First, the sample size was small, and the statistical power was limited. Second, the follow-up period was relatively short. Third, the criteria for the bending angle of the body in patients with PISA+ were small and did not equal the recent diagnostic criteria for PS [16]. Fourth, patients with dementia or mild cognitive impairment were excluded; therefore, we did not include those with severe lateral bending. Fifth, we did not examine retinal changes using corneal confocal microscopy, which reportedly aids in identifying neurodegeneration in patients with PD [42]. Finally, we did not evaluate the tone of the paravertebral muscles.

Nevertheless, this study reveals the biomarkers and pathophysiology of early lateral bending related to visual cognition. Early recognition of abnormal truncal bending may be beneficial for preventing severe bending. Besides anti-Parkinsonian drugs and physical therapy, further research on treatment strategies targeting visual cognitive function through the activation of parietal supramarginal gyrus, cuneus, or precuneus and visual training is warranted.

## 5. Conclusions

In the presymptomatic stage of lateral bending in patients with PD, there was decreased perfusion in the cortex relative to the visual cortex (including the orbitofrontal cortex and supramarginal gyrus) and impairment of spatial cognition. In addition, reduced saccades, slow and small microsaccades, and decreased blood flow in precuneus and cuneus are observed when patients experience lateral bending. Cost-effective screening for PS at the presymptomatic stage of PS includes stability and position discrimination tests.

## Figures and Tables

**Table 1:** Clinical findings of patients with Parkinson's disease at enrollment.

		PISA-*N* = 30	PISA+ *N* = 20	*p*
Age of onset		65.3 ± 9.1	64.1 ± 8.0	>0.05
Disease duration	Years	5.6 ± 4.3	6.9 ± 7.8	>0.05
Sex	Male : female	12 : 18	11 : 09	>0.05
Levodopa	mg/day	253.5 ± 183.7	359.5 ± 159.4	**<0.05**
Gravicorder				
Length-open		69.3 ± 38.6	121.0 ± 56.8	**<0.0001**
Length-close		80.4 ± 40.0	130.8 ± 76.1	**<0.001**
Area-open		4.5 ± 3.8	7.7 ± 4.6	**<0.001**
Area-close		5.1 ± 3.5	6.5 ± 5.3	>0.05
Romberg rate-area		1.4 ± 0.8	0.9 ± 0.6	**<0.05**
MMSE		28.6 ± 1.4	28.6 ± 1.5	>0.05
Moca-J		24.1 ± 3.6	23.3 ± 2.6	>0.05
Position discrimination		19.1 ± 1.0	17.8 ± 3.3	>0.05
WAIS III blocking		31.0 ± 12.7	26.3 ± 6.7	>0.05
MDS-UPDRS Part III		22.3 ± 12.3	23.5 ± 10.4	>0.05
Microsaccade	*n*	12.6 ± 10.1	9.9 ± 11.0	>0.05
Microsaccade amplitude	Degree	0.43 ± 0.57	0.24 ± 0.17	>0.05
Microsaccade velocity	Degree/msec	54.4 ± 86.6	28.6 ± 19.8	>0.05
Number of saccade in AOI		0.32 ± 1.22	0.41 ± 1.58	>0.05
Pupil diameter	Degree	2.9 ± 0.3	3.2 ± 0.5	>0.05

MMSE: Mini-Mental State Examination, Moca-J: Montreal Cognitive Assessment Japanese version, WAIS-III: Wechsler Adult Intelligence Scale-Third Edition, MDS-UPDRS: Movement Disorder Society Unified Parkinson's Disease Rating Scale, and AOI: area of interest. Bold indicates a significant difference of *P* < 0.05 or more.

**Table 2:** Clinical findings at enrollment of patients divided into three groups according to the course of PISA after 2-year follow-up.

		PISA--	PISA-+	PISA++	PISA--vs. PISA-+	PISA-+ vs. PISA++	PISA--vs. PISA++
*n*		21	9	20	*p*	*p*	*p*
Onset age		65.9 ± 10.0	65.1 ± 7.3	62.9 ± 8.5	>0.05	>0.05	>0.05
Disease duration	Years	4.6 ± 3.7	7.8 ± 4.8	6.8 ± 2.7	>0.05	>0.05	**0.023**
Levodopa daily dose	mg/day	216.7 ± 149.4	361.1 ± 224.7	333.3 ± 178.9	**0.049**	>0.05	**0.007**
Dopamine agonist levodopa equivalent dose	mg/day	74.5 ± 100.3	51.1 ± 72.9	135.6 ± 87.8	>0.05	>0.05	>0.05
Total levodopa equivalent dose	mg/day	324.5 ± 201.8	457.4 ± 244.7	574.9 ± 252.6	>0.05	>0.05	**0.003**
Gravicorder							
Length-open	cm	67.7 ± 39.0	87.6 ± 49.9	120.6 ± 63.3	>0.05	>0.05	**0.001**
Length-close	cm	76.6 ± 39.0	105.6 ± 52.1	131.2 ± 87.4	>0.05	>0.05	**0.009**
Romberg rate-Length		1.20 ± 0.24	1.42 ± 0.70	1.05 ± 0.70	>0.05	**0.032**	>0.05
Area-open	cm^2^	4.10 ± 2.72	6.75 ± 6.00	7.80 ± 4.9	>0.05	>0.05	**0.005**
Area-close	cm^2^	4.71 ± 3.14	7.64 ± 5.65	5.3 ± 2.8	>0.05	>0.05	>0.05
Romberg rate-area		1.34 ± 0.76	1.50 ± 1.07	0.73 ± 0.28	>0.05	**0.007**	**0.025**
MMSE		28.8 ± 1.3	27.3 ± 1.9	28.9 ± 1.0	>0.05	**0.011**	>0.05
Moca-J		24.7 ± 3.8	22.2 ± 2.9	23.9 ± 2.4	>0.05	>0.05	>0.05
Dot counting		10.3 ± 1.3	10.0 ± 0.0	9.7 ± 0.6	>0.05	>0.05	>0.05
Position discrimination		19.4 ± 0.7	17.7 ± 1.4	17.9 ± 1.4	**0.001**	>0.05	>0.05
Cube analysis		9.3 ± 0.9	9.0 ± 1.3	9.4 ± 0.5	>0.05	>0.05	>0.05
Line orientation (RBANS)		15.8 ± 2.9	14.7 ± 4.0	16.4 ± 2.1	>0.05	>0.05	>0.05
Clock drawing test (Freedman)		14.0 ± 4.1	13.8 ± 1.5	14.1 ± 1.4	>0.05	>0.05	>0.05
WAIS-III blocking		32.5 ± 12.3	23.8 ± 5.1	26.7 ± 5.8	**0.035**	>0.05	>0.05
MDS-UPDRS Part III		22.9 ± 14.1	23.6 ± 9.3	23.3 ± 9.1	>0.05	>0.05	>0.05
Microsaccade	*n*	11.8 ± 10.9	14.9 ± 8.7	11.1 ± 11.9	>0.05	>0.05	>0.05
Microsaccade amplitude	Degree	0.47 ± 0.71	0.36 ± 0.10	0.24 ± 0.17	>0.05	**0.026**	>0.05
Microsaccade velocity	Degree/msec	61.83 ± 107.33	41.24 ± 8.29	28.28 ± 19.00	>0.05	**0.017**	>0.05
Number of saccade in AOI	*n*	0.34 ± 1.28	0.25 ± 1.0	0.41 ± 1.6	>0.05	**0.018**	>0.05
Average duration of fixations	msec	294.3 ± 217.9	451.0 ± 323.8	277.0 ± 152.7	**<0.001**	**<0.001**	**0.044**
Time to entry saccade	msec	47612.1 ± 48993.9	52996.4 ± 55601.5	50976.0 ± 53323.2	>0.05	>0.05	>0.05
Pupil diameter	Degree	2.90 ± 0.30	2.85 ± 0.54	3.16 ± 0.42	>0.05	>0.05	>0.05

MMSE: Mini-Mental State Examination, Moca-J: Montreal Cognitive Assessment Japanese version, RBANS: Repeatable Battery for the Assessment of Neuropsychological Status, WAIS-III: Wechsler Adult Intelligence Scale-Third Edition, MDS-UPDRS: Movement Disorder Society Unified Parkinson's Disease Rating Scale, AOI: area of interest, PISA++: patients who had PISA+ at enrollment, PISA-+: patients without PISA+ that developed PISA+ during the 2-year period, and PISA--: patients without PISA+ that did not develop PISA+ during the 2-year period. Bold indicates a significant difference of *P* < 0.05 or more.

## Data Availability

The clinical data for this study are available from the corresponding author upon reasonable request.
